# Polymorphism FXII 46C>T and cardiovascular risk: additional data from Spanish and Tunisian patients

**DOI:** 10.1186/1756-0500-2-154

**Published:** 2009-07-31

**Authors:** Georgios Athanasiadis, Esther Esteban, Magdanela Gayà Vidal, Robert Carreras Torres, Raoudha Bahri, Pedro Moral

**Affiliations:** 1 Unitat d'Antropologia, Facultat de Biologia, Universitat de Barcelona, Barcelona, Spain; 2 Faculté de Pharmacie de Monastir, Université de Monastir, Monastir, Tunisia

## Abstract

**Background:**

Previous studies showed an association between Coagulation Factor XII 46C>T polymorphism and variation in FXII plasma levels, as 46C>T seems to affect the translation efficiency. Case-control studies in Spanish samples indicated that genotype T/T is an independent risk factor for venous thrombosis, ischemic stroke and acute coronary artery disease. In this study, we tried to reaffirm the importance of 46C>T in two samples from Spain and Tunisia.

**Findings:**

A Transmission Disequilibrium Test (TDT) based on 101 family trios from Barcelona with one offspring affected by ischemic heart disease and a classical case-control study based on 76 patients with IHD and 118 healthy individuals from North and Centre-South Tunisia were conducted. Subjects were genotyped for 46C>T and data were analyzed accordingly, revealing no association in any of the two samples (TDT: P = 0.16, relative risk 1.17; case-control study: P = 0.59, odds ratio 1.36).

**Conclusion:**

The results suggest that 46C>T is not a risk factor for ischemic heart disease in any of the two analyzed samples and therefore the polymorphism seems not to be a universal risk factor for cardiovascular diseases.

## Findings

The gene of coagulation Factor (F) XVII has been repeatedly analysed in many epidemiological studies on cardiovascular (CV) genetic risk with controversial results. It has been reported that FXII plasma levels exhibit a 67% of heritability and that these levels are strongly determined by F12 gene variants.[1] Although low FXII plasma levels have been reported to affect the development of CV diseases, it has been recently proposed that, rather than the cause of thrombosis, these low levels are actually the result of it. [2,3]

A highlighted F12 polymorphism is the 46C>T transition. It seems that 46C>T affects the translation efficiency, leading to reduced FXII plasma levels.[4] This polymorphism (with allele frequencies 0.8/0.2 in Caucasians) accounts for 40% of the variance in FVII activity in a Spanish Mediterranean population and seems to fit a log additive model of inheritance with an allele dose-dependent effect.[1,4]

Previous genetic association studies between 46C>T and CV risk showed that genotype T/T increases significantly the risk for venous thrombosis, ischemic stroke and acute coronary artery disease in a Spanish population. [5-7] The odds ratio in acute coronary artery disease was estimated around 4.8.[7] Conversely, there are also studies that either do not detect genetic association between this genotype and CV risk or alternatively report association between the C/C genotype and CV risk.[2,8]

In this context, this study provides additional data about the allele frequency distribution of 46C>T in two new Mediterranean samples. These data are used in an attempt to confirm previous genetic association studies, by a double epidemiological design: a family-based analysis (Transmission Disequilibrium Test - TDT) applied in a Spanish sample and a classical case-control study applied in a Tunisian sample.

A total of 101 nuclear families (n = 302) with one offspring (93 males and 8 females) clinically diagnosed for ischemic heart disease (IHD) were analyzed, all from the area of Barcelona, Spain. A comprehensive description of the families, diagnosis criteria and DNA extraction methods has been reported elsewhere.[9] Moreover, 76 patients with IHD complicated by myocardial infarction, all from Monastir, Tunisia, as well as 118 unrelated healthy autochthonous individuals from North and Central-North Tunisia were also analyzed in an unmatched case-control design.[10] This study has been performed in accordance with the Ethical Committee guidelines of the participating Hospitals and Universities and all subjects participating in the study signed a written informed consent.

Polymorphism 46C>T was genotyped by Real-Time PCR, using the standard TaqMan^® ^SNP genotyping assay protocol of Applied Biosystems for a total volume of 5 μl per well. DNA amplification as well as fluorescence intensity measurements of the final reaction product and data collection were carried out with an ABI PRISM^® ^7900 HT Sequence Detection System.

Allele frequencies in the Spanish IHD patients and their parents as well as in patients and controls from Tunisia were calculated directly. A TDT analysis was performed in the IHD families from Barcelona, using the FBAT v2.0.2c program.[11] In the Tunisian case-control study, association was examined with Epi Info™ v3.4.3.[12] This program was also used for the calculation of the relative risk in the TDT analysis and the odds ratio (T/T vs. C/C and C/T) in the case-control design. Sample power was calculated using QUANTO v1.2.3 assuming a CV disease prevalence of 0.07.[13]

Frequencies of the T allele in the Spanish and Tunisian samples are shown in Table 1. In the Spanish families, the TDT analysis indicated a low relative risk of 1.17 (95% CI: 0.75 <RR<1.82) for the T allele, without any significant transmission to the patients (p = 0.16). Power calculations revealed that the minimum detectable risk our family sample size (n = 101) could find was approximately 1.9 (with a power >80%). In the Tunisian sample, the estimated odds ratio for the T/T genotype was 1.36 (95% CI: 0.36 < OR <4.93), without any significant association (p = 0.59). Again, the minimum detectable effect our set of 76 patients and 118 controls could find was around 1.9. Finally, no significant differences were found in any pairwise comparison of the data in Table 1.

**Table 1:** Allele and genotype frequencies of the 46C>T in the Spanish and Tunisian CV patients

		Allele frequencies		Genotype frequencies			
		
		C	T	CC	CT	TT	N
Spain	affected children	0.768	0.232	0.590	0.356	0.054	97
	parents	0.799	0.201	0.638	0.321	0.040	137

Tunisia	cases	0.770	0.230	0.593	0.354	0.053	76
	controls	0.750	0.250	0.563	0.375	0.063	118

Our study showed that the allele frequencies of the FXII 46C>T polymorphism are similar in the Spanish and Tunisian samples, in accordance with the Caucasian pattern. This suggests a stable presence of 46C>T in different Mediterranean regions. On the other hand, our results indicate the absence of association between 46C>T and CV disease in both samples.

The similar allele frequency distributions between Spanish patients and parents and between Tunisian patients and controls provide a first rough estimate of the lack of association. Moreover, our Tunisian sample has enough power (>99%) to detect an odds ratio of 4.8, as reported elsewhere, reinforcing the consistency of the lack of association detected.[7] As far as we know, this is the first time the potential role of 46C>T is tested through a family-based TDT analysis. Out findings confirm the negative results of the case-control design, always taking into account that our family trios have power only to detect risks above 1.9.

The diverse results of previous studies regarding association between polymorphism 46C>T and CV disease could be explained, at least in part, by the differences in study design, the definition of inclusion and exclusion criteria and the number of participants. In our case, the low sample size in the TDT study and the lack of a matched case-control design impose some caution in the interpretation of our results.

In short, our results provide indirect evidence that 46C>T is not a universal independent risk factor for CV diseases. What seems to be well established is that the T allele affects the variation of intermediate phenotypes (FXII plasma levels). If this is true, then the diversity of results in genetic association studies is hard to explain. This observation leads us to adopt the idea that the low levels of FXII are the consequence rather than the cause of the disease, as has been recently suggested.[3] This idea finds solid ground when combined with other independent results on the lack of causality between FXII deficiency and venous thrombosis. [14,15] In this context, 46C>T polymorphism may not be a direct determinant of CV risk.

## Competing interests

The authors declare that they have no competing interests.

## Authors' contributions

GA carried out the SNP genotyping, participated in the statistical analyses and wrote the original manuscript. EE gave important advice for the improvement of the manuscript. MGV carried out the Spanish sample preparation. RCT helped with the statistical analyses. RB carried out the DNA extraction from the Tunisian samples. PM participated in the design and coordination of the study and helped to draft the manuscript. All authors read and approved the final manuscript.
